# Dr. Albert H. Crawford

**Published:** 1899-02

**Authors:** 


Dr. Albert H. Crawford, of Buffalo, died suddenly at his residence, 274 East Swan street, Friday, January 20, 1899, aged 58 years. The day before his death, Dr. Crawford, apparently in his good health, started out on his bicycle to visit a patient on the Tifft Farm. When he had reached the junction of Louisiana and Ohio streets he felt a peculiar numbness stealing over him, which his professional knowledge told him meant a premonition of apoplexy. He managed to dismount from his wheel and reach the side of the street, where he sank down. Consciousness had not deserted him and he asked a woman, who was passing, to call a policeman. The officer procured a carriage and he was taken to his home. Medical aid was at once called in, but he soon lapsed into unconsciousness and quietly passed away next morning.

Dr. Crawford was a native of Corfu, Genesee County, where he passed his early life. He graduated in medicine at Buffalo University Medical College in February, 1865. He engaged in practice for a time in one of the south towns of Erie County, but afterward located in Corfu. He came to Buffalo in 1881, where he has since pursued the practice of his profession with credit to himself and in the enjoyment of the confidence of his patients and professional colleagues. He was wholly absorbed by his profession and his domestic ties. He was always faithful to his patients and enjoyed the respect of a large circle of neighbors and friends. He was a member of the Medical Union and of the Medical Society of the County of Erie. He is survived by a widow and two young daughters. His funeral was largely attended on Monday, January 23, 1899, and the interment was at Corfu.

